# Medical Papers in Public Libraries

**Published:** 1916-02-26

**Authors:** 


*To the Editor of* The Hospital.

### Medical Papers in Public Libraries.

*To the Editor of* The Hospital.

Sir,—Another addition to the "W" series of public libraries showing The Hospital might be found in the Wandsworth Borough Library at Streatham. It was there, some six years ago, that I first saw your paper, and I soon found the need for my own copy, and I have not missed a copy since. Having to leave the Metropolis for a lonely and remote station in Devonshire, I now look forward even more eagerly to every Friday evening, which brings The Hospital with its news and help.—Yours faithfully,

An Occasional Contributor.

February 20, 1916.

